# Skin Fibroblasts from Patients with Type 1 Diabetes (T1D) Can Be Chemically Transdifferentiated into Insulin-Expressing Clusters: A Transgene-Free Approach

**DOI:** 10.1371/journal.pone.0100369

**Published:** 2014-06-25

**Authors:** Federico Pereyra-Bonnet, María L. Gimeno, Nelson R. Argumedo, Marcelo Ielpi, Johana A. Cardozo, Carla A. Giménez, Sung-Ho Hyon, Marta Balzaretti, Mónica Loresi, Patricia Fainstein-Day, León E. Litwak, Pablo F. Argibay

**Affiliations:** 1 Instituto de Ciencias Básicas y Medicina Experimental (ICBME), Hospital Italiano de Buenos Aires (HIBA), Buenos Aires, Argentina; 2 General Surgery Service, HIBA, Buenos Aires, Argentina; 3 Endocrinology and Nuclear Medicine Service, HIBA, Buenos Aires, Argentina; Université Paris Descartes, France

## Abstract

The conversion of differentiated cells into insulin-producing cells is a promising approach for the autologous replacement of pancreatic cells in patients with type 1 diabetes (T1D). At present, cellular reprogramming strategies encompass ethical problems, epigenetic failure or teratoma formation, which has prompted the development of new approaches. Here, we report a novel technique for the conversion of skin fibroblasts from T1D patients into insulin-expressing clusters using only drug-based induction. Our results demonstrate that skin fibroblasts from diabetic patients have pancreatic differentiation capacities and avoid the necessity of using transgenic strategies, stem cell sources or global demethylation steps. These findings open new possibilities for studying diabetes mechanisms, drug screenings and ultimately autologous transgenic-free regenerative medicine therapies in patients with T1D.

## Introduction

Type 1 diabetes (T1D), which is a multifactorial autoimmune disease that is caused by β-cell destruction that leads to the deregulation of glucose homeostasis, has initially been treated by exogenous insulin therapy or through cellular replacement by a whole-pancreas transplant or by islet transplantation into the portal vein [1]. However, the low ratio of donors to recipients for cellular replacement requires more viable alternative treatments.

At present, it is of special interest for the diabetes community to find a way to produce *ex vivo* pancreatic cell masses to restore biological functions that are lost due to cellular deficits. Because T1D only affects a single cell type, T1D can be treated with novel cellular replacement therapies that are based on reprogramming human embryonic stem cells (hESC) [2] or with human induced pluripotent stem cells (hiPSC) [3], [4] into pancreatic-like cells. However, hESC and hiPSC show several disadvantages, such as ethical problems [5], transgenic strategies [6] or epigenetic failure [7], which limit their use to in vitro assays or preclinical models [8]. Currently, the need for finding new strategies that are based on reprogrammed cells for safer replacement therapies remains a major concern.

Transdifferentiation is the direct conversion of one somatic cell type into another type without passing through pluripotential states [9]. The transdifferentiation of patients' fibroblasts into pancreatic-like cells could render a much more straightforward clinical application of reprogrammed cells based therapy without the main concerns of hESC and hiPSC. The most commonly used methods for transdifferentiation consider genetic modification strategies as viral vectors, which encompass several clinical risks, such as teratoma formation [10].

Fibroblast transdifferentiation by chemicals has been demonstrated in in vivo and in vitro cell lineages, e.g., osteocytes [11]–[17], condrocytes [18], [19], adipocytes [20], [21] and hepatocytes [22]. Surprisingly, skin fibroblasts were also chemically reprogrammed toward a pluripotent state, demonstrating its extreme plasticity [23], [24]. Recently, insulin-secreting cells have been transdifferentiated from adult human skin fibroblasts using chemicals with a global demethylation step [25]. This is a provocative approach, however the demethylation step could be perceived as an unsafe procedure from a clinical point of view. In addition, chemical transdifferentiation to pancreatic linage has never been explored in T1D patient-specific autologous fibroblasts. Fibroblasts that are chemically transdifferentiated from diabetic patients present several advantages, such as containing the disease-associated genotype pattern for disease modeling; avoiding immunological rejection for future cell therapies, and are a unique opportunity to test whether these cells can be transdifferentiated into the desired pancreatic phenotype.

Our proof-of-principle study shows that the transdifferentiation of skin fibroblasts from patients with T1D toward insulin-producing clusters can be manipulated using only chemical compounds avoiding the necessity of use global demethylation steps. Changing the identity of fibroblasts from diabetic patients without using genetic modifications or demethylation step would be a safer and more reliable clinical approach.

## Materials and Methods

### Ethics statement

Protocols and informed consent were approved by the Institutional Ethics Committee (Institutional Ethics Committee of Research Protocols HIBA; Resolution CEPI 1672). All donors provided written informed consent for the collection of all samples and subsequent analysis. Animal experiments were approved by the Institutional Animal Care and Use Committee (Experimental Research Committee HIBA; Resolution E/84).

### Isolation and culture of skin fibroblasts

Skin biopsies were obtained from two T1D patients (HF1 and HF2; Table 1) and one healthy volunteer (HF0). The skin biopsies were cultured and expanded in fibroblast medium (FM: DMEM-Knockout with 10% FBS and 1% antibiotics) at 37°C in 5% CO_2_ and frozen in FM containing 10% DMSO at passage 3. The characterization of the initial fibroblast culture was performed by microarrays and immunocytochemistry, as described below.

**Table 1:** Patients' demographic and clinical characteristics.

Patient with T1D	Age/gender	Diagnosed at age of	Family history	Subcutaneous Insulin daily dosage	Hemoglobin A1C
HF1	59/female	9	None	42U	6.7%
HF2	18/male	17	None	40U	9.5%

T1D: Type 1 Diabetes.

HF: human fibroblasts.

### Chemical transdifferentiation

In total, 1.5x10^5^ fibroblast cells were seeded per well in a six-well plate in DMEM, 10% SFB and 2% antibiotics. Fibroblasts were enzymatically passaged using a xeno-free trypsin-like enzyme (Invitrogen, Carlsbad, CA, USA). Two days later, the medium was replaced with transdifferentiation medium [TM: 40 ng/mL bFGF, 20% xeno-free serum replacement (Invitrogen), 2 mM glutamine, 0.1 mM nonessential amino acids, 0.1 mM β-mercaptoethanol, and 2% antibiotics in DMEM/F12 Knockout]. After 4 days, the TM was changed and maintained for 3 more days. Then, the medium was replaced with TM containing 10 mM nicotinamide (Sigma, St Louis, MO, USA). The medium was changed after 4 days and maintained for 3 more days. The medium was then replaced with TM containing 50 ng/mL exendine-4 (Ex-4, Baxter, Indiana, USA). The medium was changed after 4 days and maintained for 3 more days. At this time, culture cells were supplemented with cocktail 1 [50 ng/mL Ex-4, 1% B27, 2% insulin-transferrin-telenium-A (ITS, Invitrogen), 5 µg/mL human insulin, 25 ng/mL IGF1, 22 mM glucose] and cultured for an additional 4-day period. Cells were replated onto CellStar xeno-free (Invitrogen) coated six-well plates in TM-containing cocktail 2 [50 ng/mL Ex-4, 2% ITS, 5 µg/mL human insulin, and 22 mM glucose] and cultured for 3 days. We used parental fibroblasts that were cultured in FM for 2–5 days as untreated cells or that were cultured in FM for 30 days as treated control cells.

### Gene expression analysis

Total RNA was isolated with an MicroKit (Qiagen, Valencia, CA). For RT-PCR and qPCR, RNA was reverse-transcribed using Superscript III. Specific intron-spanning primers were used in the PCR and qPCR (see Table S1 in File S1). The number of PCR cycles ranged from 35–38. The qPCR was performed using a LightCycler FastStart DNA Master SYBR Green Kit (Roche, Mannhein, Germany), and β-actin gene expression was used to standardize gene expression levels. All experiments were repeated at least twice. For whole genome expression analysis, Illumina HumanHT-12 v3.0 Expression Beadchip mRNA arrays (RefSeq-38) were performed in transdifferentiated and untreated cells by Macrogen (Macrogen Inc, Korea).

### In vitro immunocytochemistry

Cell cultures were fixed with 4% of paraformaldehyde solution in phosphate buffer for 10 min, followed by a phosphate buffered saline (PBS) wash, pH 7.2, and blocked for 5 minutes in Power Block (Biogenex San Ramon, USA). The primary antibodies were incubated at 4°C overnight, and the secondary antibody was incubated for 1 hour at room temperature. The following primary antibodies were used: mouse anti-human vimentin (Chemicon, CBL202), CD90 Thy-1 (Santa Cruz, H-110) and rabbit anti-glucagon polyclonal (Linco, 4031). The secondary antibody was MULTILINK (Biogenex, HK268). Texas Red streptavidin (Vector, SA-5006) was added for 1 hour at room temperature, and nuclei were counterstained with Höescht staining. Imaging was performed using an epifluorescence microscope (Nikon, Eclipse E400).

### C-peptide detection

For C-peptide detection, total protein was obtained from the cells using cold lysis buffer with a protease inhibitor cocktail. Protein concentration was determined using a Bio-Rad Protein Assay kit. C-peptide measurements were determined by a solid phase, two-site chemiluminescent immunoassay (Siemens).

### Lipofection

To demonstrate the transdifferentiation nature of our protocol, (without passing through the pluripotent state), the treated cells were lipofected (Lipofectamine 2000, Invitrogen) with the GOF18-EGFP plasmid (0.4 µg/3x10^5^ cells), which contains GFP under *OCT4* promoter control at day 15.

### Methylation status in the promoter genes

Genomic DNA of transdifferentiated and untreated cells, as well as human pancreatic islets, was purified using a MiniKit column (Qiagen) and treated using an bisulfite kit (Qiagen). The post-bisulfite promoter region of the human *PDX1* (ID3651), *OCT-4* (ID 5460) and *NANOG* (ID 79923) genes were amplified by PCR using specific primers (see Table S1 in File S1). The PCR products were purified and direct sequenced by Macrogen (Macrogen Inc). For global DNA methylation analyses we used LINE 1 as a marker [26]–[28]. The degree of methylation was expressed as percentage of methylated cytosines over the sum of methylated and unmethylated cytosines.

### Cytogenetic and DNA fingerprinting analysis

Karyotyping was performed on G-banded metaphase cells by Primagen Lab (Buenos Aires, Argentina). Short tandem repeat (STR) analysis was performed in 9 standard polymorphic DNA loci between parental and transdifferentiated cells.

### Engraftment of mice with transdifferentiated fibroblasts

Nude mice were transplanted intrapancreatically with 3-9x10^5^ transdifferentiated cells (n = 6), PBS (sham; n = 4) or 30-day fibroblasts were transplanted as a negative control (n = 4). Mice were anaesthetized, and cells were microinjected with 50 µl of PBS into the splenic portion of the pancreas. At 15–16 days post-transplantation, the mice received an intraperitoneal injection of 70 mg STZ per kg body weight for 5 consecutive days. To determine the survival and presence of the transplanted cells in the mouse pancreas, PCR and RT-PCR were performed as described above (see Table S1 in File S1).

### Glucose-stimulated insulin secretion

Transplantation function was assessed by performing measurements of human insulin serum in response to an intraperitoneal injection of a 30% glucose solution at a dose of 3 g/kg body weight. Blood was collected from individuals in the basal state and at 30 min after glucose administration, before and after STZ-induced diabetes. Insulin was determined by a one-step chemiluminescent immunometric assay using an ARCHITECT i2000SR (ABBOTT). Tail blood glucose measurements were taken with glucometer strips (Accu-Check, Aviva).

### Statistical analysis

Statistical comparisons of the obtained results were performed using the Fisher test in RT-PCR, Student's *t*-test in qPCR and methylation analysis and an ANOVA for in vivo results. For microarrays, raw data were analyzed using the software Illumina GenomeStudio v2011.1, and the statistical significance of the expression data was determined using the fold-change. Hierarchical cluster analysis was performed using complete linkage, and Euclidean distance was used as a measure of similarity. Gene enrichment and functional annotation analysis for a significant probe list was performed using the DAVID database (http://david.abcc.ncifcrf.gov/home.jsp). All data analyses and the visualization of differentially expressed genes were conducted using the R 2.14.0 system (www.r-project.org). Differences were considered statistically significant when *P*<0.05. The data are shown as averages and s.d or s.e.m.

## Results

### Characterization of the initial fibroblast cell culture

**Figure 1:**
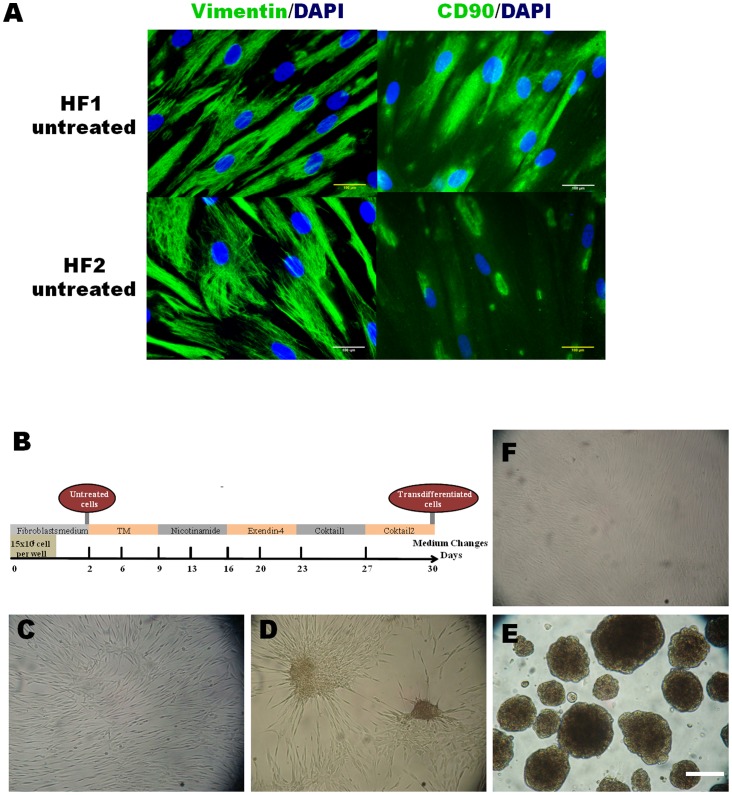
Original fibroblasts population and chemical transdifferentiation. (**A**) Characterization of the original fibroblast population in the initial cell cultures by immunocytochemistry. (**B**) Time schedule of chemical transdifferentiation protocol. (**C**) Morphology of chemically treated fibroblast obtained from a type 1 diabetes patient at day 12. (**D**) Typical image of treated cells at day 24. The cells migrate to the surface of the petri dish and begin to form islet-like clusters. (**E**) Typical image of the transdifferentiated cells with clusters morphology obtained by the end of the treatment (day 30). (**F**) Control treated fibroblast without chemical induction. ***HF1 and HF2***: fibroblasts from patients with type 1 diabetes. ***Transdiferentiated***: fibroblasts chemically transdifferentiated for 30 days; ***Untreated***: fibroblasts day 2–5; ***Control treated***: fibroblasts cultured for 30 days without chemical induction (Bar 200 µm). ***TM***: Transdifferentiation Medium.

We attempted to characterize the starting fibroblast culture using microarray data and immunocytochemistry to verify the lineage identity before the chemical transdifferentiation process begins. We initially found that this population expressed the fibroblast cell-surface marker CD106 and two differentially expressed fibroblast markers, CD10 and CD26, with respect to mesenchymal stromal cells [29]. Additionally, this population did not contain cells that expressed SSE3, a multilineage-differentiating marker for induced pluripotent stem (iPS) cells from human fibroblasts [30]. Fibroblasts likewise expressed genes of the extracellular matrix and cytoskeleton, which are proper for a fibroblast phenotype (Table 2). To more rigorously characterize these initial fibroblast populations, we examined primary fibroblast lineage markers by immunocytochemistry. The fibroblasts expressed vimentin, which is a marker to distinguish their mesodermal origin, and CD90, which is a marker of cellular fibronectin in human endothelial cells (Fig. 1A).

**Table 2:** Characterizations of the original fibroblast population in the star cell cultures by microarrays.

Category	Gene	HF1		HF2	
		Signal	*P* value	Signal	*P* value
***Cell surface***	CD10 (MME)	13911.07	**0**	13555.11	**0**
	CD26 (DPP4)	10428.74	**0**	2720.099	**0**
	CD151 (SFA1)	42596.34	**0**	24698.63	**0**
	CD106 (VCAM1)	319.2572	**0**	379.1841	**0**
	CD45 (PTPRC)	169.3357	**0.450649**	177.5038	**0.368831**
	SSE3 (B3GALT5)	123.3048	**0.784416**	127.1248	**0.78052**
***Extracellular matrix***	COL8A1	2480.151	**0**	16653.33	**0**
	COL5A2	10972.07	**0**	25406.67	**0**
	COL1A1	39191.43	**0**	51123.93	**0**
	COL1A2	62232.02	**0**	61975.48	**0**
	COL3A1	32437.5	**0**	45592.39	**0**
	FMOD	759.7853	**0**	5329.169	**0**
	ELN	884.2727	**0**	1655.42	**0**
	TNC	7137.219	**0**	9910.19	**0**
	LAMB2	8506.021	**0**	9031.922	**0**
	LAMB3	260.1513	**0**	232.3912	**0**
***Cytoskeleton***	VIM	60390.57	**0**	50957.55	**0**
	FSP1 (S100A4)	20917.76	**0**	12413.17	**0**

### The chemical induction modified the cell morphology from fibroblasts to insulin-producing clusters

We have developed a 30-day protocol that includes the incorporation of drug cocktails consecutively to accomplish the in vitro transdifferentiation into pancreatic-like cells (Fig. 1B). In the first step of the protocol, isolated fibroblasts were transferred to the transdifferentiation medium, which was enriched with bFGF. We reasoned that the addition of bFGF might allow the development of pancreatic endoderm because this molecule is produced by the notochord during embryonic dorsal pancreas development [31]. As a variant of this strategy, we decided to use a 10-fold increased concentration (40 ng/ml) of bFGF, compared with traditional protocols (4 ng/ml), to supplement any disability of differentiation capacity from fibroblasts compared with stem cells. Activin A, which is a member of the transforming growth factor-β (TGF-β) superfamily, has been shown to induce endodermal differentiation. However, activin A promotes not only endoderm development but also the formation of neuronal cells. For this reason, the application of activin A was avoided in our chemical treatment. In a second step, we added the poly-synthase (ADP-ribose) inhibitor nicotinamide, expecting a clearly differentiated and increased β-cell mass. At approximately day 12 after induction, some cell conglomerates became visible at the center of the plate (Fig. 1C). In the third step, exedin-4 was included to further promote the differentiation of the definitive endoderm to a pancreatic lineage by enhancing *PDX1* gene expression [32]. At the end of the third step (day 24), colonies were observed all over the plate (Fig. 1D). In the fourth step, other components of the drug cocktail, such as IGF1, B27 and insulin, were included to induce the further maturation of pancreatic endocrine cells. In addition, it has been reported that three-dimensional aggregate formation was necessary to generate insulin-producing cells. Therefore, we decided to include an extracellular matrix (Matrigel) to support the tridimensional growth of the transdifferentiated cells. At day 30, cell colonies detached from the Petri dish surface and formed clusters (Fig. 1E). By the end of the treatment, all treated cells in the plate displayed the cluster morphology (for patients with T1D: HF1-HF2 and for the healthier volunteer: HF0). None of these characteristics were detected in untreated or treated control cells (Fig. 1F). Another improvement of this protocol was the utilization of xeno-free medium (devoid of animal products) in the chemical induction medium. Cell culture in presence of animal products poses a potential risk for incorporating animal pathogens to human cells [33] and could imply a barrier by regulatory agencies in the future. A xeno-free induction protocol is a significant step toward the translation of this technology from the laboratory to the clinic.

### The chemical induction activates the transcription of numerous pancreatic genes, downregulates the expression of fibroblasts transcripts and does not activate embryonic cell markers in vitro

**Figure 2:**
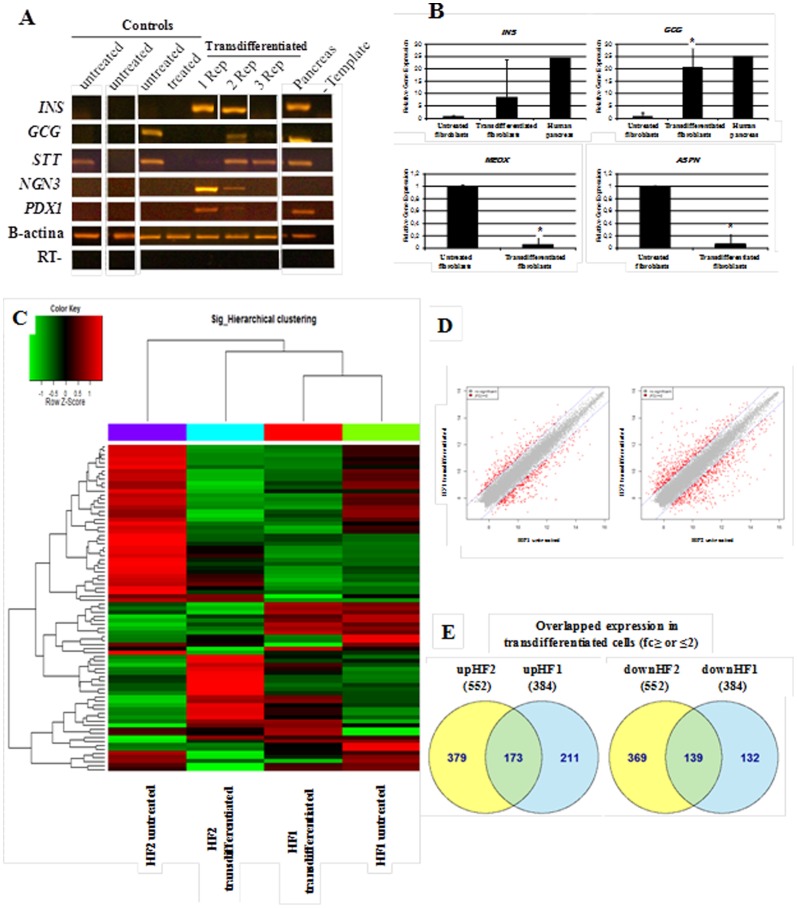
Gene expression analyses of fibroblasts before and after chemical transdifferentiation. (**A**) Gene-expression profiles of transdifferentiated in vitro fibroblasts from patient whit diabetes type 1 (patient HF1, tree replicates) and parental controls fibroblasts by RT-PCR analysis. (**B**) Gene-expression profiles of transdifferentiated fibroblasts (n = 6 replicates) and untreated control fibroblasts (n = 3 replicates) by quantitative PCR analysis in pancreatic genes (*INS*, *GCG*) and fibroblast markers (*MEOX*, *ASPN*) (*t* test, P<0.05). The bars represent the standard errors of independent experiments. (**C**) Hierarchical clustering of differentially expressed genes. Using normalized value, plotted a heat map of hierarchical clustering on distance similarity for samples and probes. (**D**) Plotted a scatter plot of expression level between groups. The red dots indicate significant probes (2-Fold). (**E**) Venn diagram for overlapped upregulated (up) and downregulated (down) gene expression in transdifferentiated cells groups. ***HF1 and HF2***: fibroblasts from patients with type 1 diabetes; ***Transdifferentiated***: fibroblasts chemically transdifferentiated for 30 days; ***Untreated***: fibroblasts day 2–5; ***Control treated***: fibroblasts cultured for 30 days without chemical induction. ***Rep***: Replicates.

A first step in the characterization of transdifferentiated cells is the identification of changes in the expression of several lineage-specific markers. For example, the markers of the target lineage-cell must be upregulated, whereas the donor cell markers must be downregulated. The RT-PCR analysis of the transdifferentiated cells in vitro from HF0, HF1 and HF2, revealed the expression of the essential pancreatic transcription factors and genes: *INS*, *GCG*, *STT*, *NGN3* and *PDX1* in 28.5% (2/7), 71.4% (5/7), 100% (7/7), 57.1% (4/7) and 42.8% (3/7) of the replicates, respectively, compared with the control groups (*P*<0.05) (Fig. 2A). In addition, we determined the expression of *PAX4*, *GLUT2* and *ISL-1* in transdifferentiated cells (data not shown). To quantify and validate these observations, we performed qPCR for *GCG* and *INS* genes, in untreated and transdifferentiated cells groups. The qPCR analysis showed that, in transdifferentiated cells from diabetic and the healthy donor, *GCG* and *INS* were upregulated, and *ASPN* and *MEOX*, which are two genes that are related to fibroblasts, were both downregulated by more than 10-fold (Fig. 2B).

The global microarrays gene expression in vitro analysis allowed us to perform a heat map of hierarchical clustering and a dendrogram that arranges the clustered samples in terms of their similarity to each other (Fig. 2C). We observed that our novel chemical protocol generates modifications on a transcriptome-wide scale and that the transdifferentiated HF1 cells were more similar to transdifferentiated HF2 cells than to their untreated parental fibroblasts, which suggested a common cell fate direction. In addition, we confirmed that 655 and 1060 genes were upregulated or downregulated, respectively, by more than 2-fold in transdifferentiated cells compared with untreated fibroblasts (for HF1 and HF2, respectively) (Fig. 2D). Additionally, 30–50% of these genes were overlapped between treated cells (Fig. 2E). Some of the top 30 overlapped and upregulated genes were directly related to the pancreatic lineage, such as *INSIG1*, *NKX2.2*, *LOC651872*, *TGM2*, *TGF*-b ligands, and Nestin, or were involved in chromatin remodeling, such as *BMPs* and *SMADs*. Likewise, some of the top 30 overlapped and downregulated genes were related to the fibroblasts lineage markers, such as *CD34*, Elastin, Filamin-B, *COL12A1* and *COL8A1*. In addition, none of expressing genes matched the typical embryonic pluripotencial cells markers (i.e., *OCT4*, *SOX2, LIN28, REX1* and *NANOG*) (see File S2). Therefore, to demonstrate the transdifferentiation nature of our protocol, (without passing through the pluripotent state) the treated cells were lipofected with the GOF18-EGFP plasmid, and as a result, no-expressing GFP cells were found showing the transdifferentiation nature of our protocol.

### Fibroblasts display a hypomethylation status before and after chemical treatment

**Figure 3:**
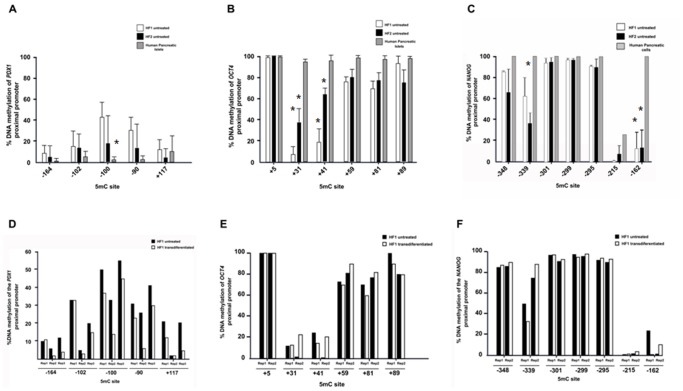
Methylation analysis of *PDX1*, *OCT4* and *NANOG* proximal promoters in fibroblasts before and after chemical transdifferentiation. (**A-C**) Hypomethylation of *PDX1*, *OCT4* and *NANOG* proximal promoters in untreated skin fibroblasts from diabetic patients (HF1 and HF2). Lack of expression of these genes was found for untreated fibroblasts. The bars represents the average and standard deviations of three (*PDX1*) and two (*OCT4* and *NANOG*) independent sequencing procedures. (**D**) Direct sequencing analysis for two *PDX*+ reprogrammed cell lines from the same diabetic patient (HF1). No tendency were found on *OCT4* (**E**) and *NANOG* (**F**) proximal promoters. **5mC site**: 5 methylation Citocine; **Rep**: Repetition.

The methylation status of the DNA sequence near the regulatory regions of genes, particularly on cytosine guanine dinucleotides (CpG) or on 5-methylcytosine (5mC), may reflect the transcription potential of these genes. The DNA methylation status in untreated fibroblasts and in chemically-treated *in vitro* cells (replicates *PDX1+* detected by RT-PCR) was observed in transcription factor binding zones that were proximal to the pancreatic lineage marker *PDX1* and the pluripotency-associated genes *OCT4* and *NANOG*. We found provocative scenarios of methylation patterns: for the *PDX1* gene promoter, most of the 5mCs that were analyzed were incompletely methylated in untreated fibroblasts, which was similar to the human pancreas (Range 10–60%; see Fig. 3A). Additionally, *OCT4* and *NANOG* were hypomethylated in untreated fibroblasts in comparison with pancreatic-committed human cells (*P*<0.05) (Fig. 3B, C). In contrast, in chemically transdifferentiated fibroblasts, we observed a *PDX1* hypomethylation trend (range 5–20%) in the 5mC -100, -90 and +117 compared with untreated fibroblasts (Fig. 3D), which coincides with the *PDX1* gene expression that was detected by RT-PCR. These 5mC bases coincide with the USF1 binding site, which is a key transcription factor of *PDX1* expression. In the cases of *OCT4* and *NANOG* gene promoters, there was not a hypomethylation trend between replicates for chemically transdifferentiated fibroblasts, compared with untreated cells (Fig. 3E,F). In addition, we used DNA methylation analyses of LINE-1 repeated sequences to evaluate global methylation. Due to the heavy methylation of repetitive elements and their distribution across the human genome (approximately 17%), this assay can detect decreases in DNA methylation and serve as a surrogate to evidence global methylation [26]–[28]. No significant changes were observed in the level of methylation after reprogramming, suggesting that our chemical treatment does not produce global DNA methylation changes (see Figure S1 in File S1).

### *In vitro* pancreatic endocrine hormones expression in transdifferentiated cells

**Figure 4:**
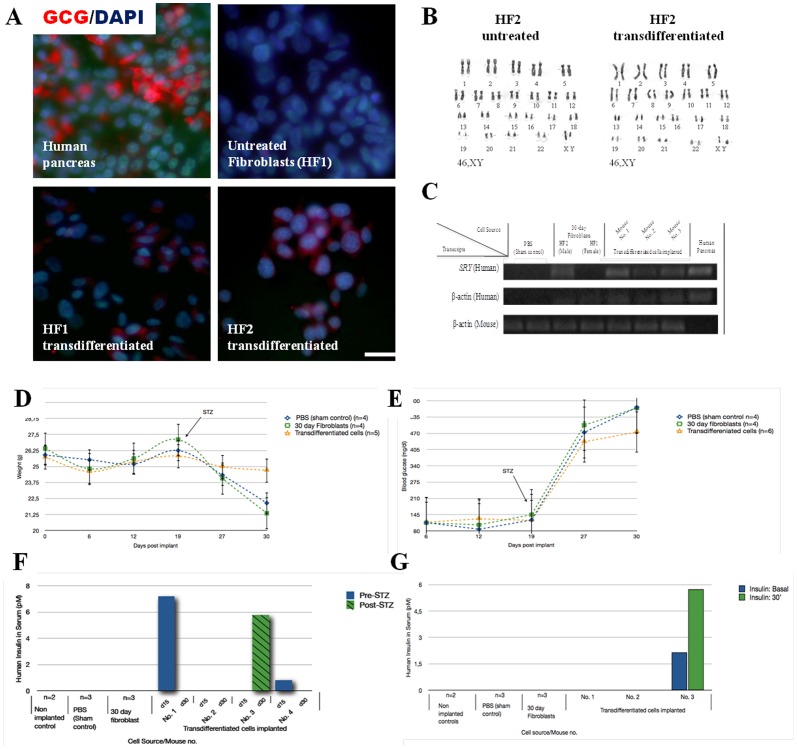
Analyses of transdifferentated cluster before and after transplantation in NUDE mouse. (**A**) Typical images of in vitro immunostaining for glucagon in human pancreas control, untreated fibroblasts and in chemically transdifferentiated fibroblasts from patient with type 1 diabetes. (**B**) G-banding karyotype in HF2 patient before and after chemical transdifferentiation treatment showing normal male karyotypes. (**C**) Survival and presence of the transplanted cells in the mouse pancreas by qPCR using specific primers for human *SRY* and β-actin genes. Analyses of streptozotocin (STZ)-treated mice that were transplanted with insulin-expressing cells transdifferentiated from fibroblasts of patients with type 1 diabetes. Mice were inspected daily for weight loss (D) and basal blood glucose levels (E). Human insulin serum was measured at 30 min after intraperitoneal glucose stimulation pre and post STZ treatment (15 and 30 day post implant) (F) and in nonfasting and 30 min after glucose stimulation at day 30 (G, glucose challenge). Error bars indicate s.d. ***HF1 and HF2***: fibroblasts from patients with type 1 diabetes; ***Transdifferentiated***: fibroblasts chemically transdifferentiated for 30 days: ***Untreated***: fibroblasts day 2–5.

Glucagon was assessed by immunocytochemical analysis and was found in 30% of the transdifferentiated fibroblasts, and no specific markers were found in untreated fibroblasts controls (Fig. 4A). Conversely, although a few untreated fibroblasts expressed the glucagon RNA marker, as determined by RT-PCR (Fig. 2A), none of these untreated fibroblasts were able to acquire glucagon expression on the protein level, which suggested the action of post transcriptional regulation. Additionally, we monitored C-peptide production to confirm the de novo synthesis of insulin by the transdifferentiated in vitro fibroblasts. C-peptide was detected (0.18 and 0.22 ng/mL) in the two transdifferentiated cells that were positive for *INS+* by RT-PCR, whereas the controls were negative. The C-peptide detection was assessed by total protein extraction after glucose stimuli. However, C-peptide was not detected in the medium in the presence of glucose (data not shown) suggesting that the cells represent an immature phenotype.

### Cytogenetic and DNA fingerprinting analysis

To exclude the possibility that transdifferentiated cells resulted from contamination with pluripotent cells that were cultured in our laboratory and/or from chromosomal abnormalities, we performed DNA fingerprinting and G-banding analysis of the in vitro cells before and after the chemical treatments. Short Tandem Repeat (STR) analysis revealed that the DNA fingerprint of transdifferentiated cells matched the genotype of the parental fibroblasts (see Table S2 in File S1), which indicated that the transdifferentiated cells were derived from fibroblasts and not from cross-contamination. In addition, the G-banding of genomic DNA revealed a normal karyotype before and after chemical treatment in HF2 (Fig. 4B), which suggested that the chemical induction did not damage the chromosome integrity in the treated cells.

### Transdifferentiated cells prevent the mice from weight loss and early hyperglycemia peaks

After the intrapancreatic transplantation of approximately 3–9x10^5^ transdifferentiated cells, mice were inspected daily for signs of pain or excessive thirst, as well as changes in basal levels of glucose and weight loss that exceeded 10% of their initial values, to determine their condition during the 30 days post-implantation. Mice were considered diabetic if their plasma glucose concentration exceeded 300 mg/dl. Because STZ toxicity toward beta cells depends on the expression of the GLUT2 glucose transporter receptor, islet-like human clusters would be protected from the cytotoxic activity of STZ due to their low expression of the transmembrane carrier protein [34], [35]. After 15 days post-STZ treatment, the nude mice that were transplanted with transdifferentiated cells did not present a significant variation in weight loss exceeding 4%, unlike the controls, Sham (15%) and 30-day fibroblasts (20%) (*P* = 0.006) (Fig. 4D). Monitoring the blood glucose levels demonstrated that endogenous mouse β-cells were destroyed by STZ, which resulted from exceeding 300 mg/dl. Although the diabetic state was not reverted by mice that were transplanted with transdifferentiated cells, these cells prevented the mice from reaching average blood glucose concentrations above 600 mg/dl, which is in contrast with the results that were observed in sham and 30-day fibroblast controls (Fig. 4E). In addition, no formation of tumor were detected, as assessed by macroscopic examination of organs and tissues in diabetized transplanted mice.

### Preliminary in vivo functionality of transdifferentiated cells

Human insulin was measured in the sera of nonfasting mice and at 30 minutes after glucose stimulation due to the evidence of increasing insulin-response to glucose administration pre- and post-STZ treatment. Mice with implanted transdifferentiated cells (mice 1, 2 and 3) showed detectable levels of human insulin (pM<200) throughout the experiment (Fig. 4F). Sham, 30-day fibroblast and non-transplanted control mice did not show detectable serum levels of insulin during the glucose challenges pre- and post-STZ. Mouse no. 2 showed a human insulin-secreting glucose-responding behavior in nonfasting and at 30-minute measurements (>2-fold increase), suggesting a moderate physiological functionality of the transdifferentiated cells upon STZ treatment (Fig. 4G). Although this result represents low levels compared with the necessary concentration of human insulin to protect the mouse from diabetes, an early detection is significant compared with the fact that the development of maximal insulin secretion requires at least 3 months post-implant [2].

### Survival and presence of the transplanted cells in the mouse pancreas

We sought to determine the survival and presence of the transplanted cells in the mouse pancreas. The qPCR assay was designed to detect human DNA over mouse DNA that was contained in a mixed-DNA sample. Species-specific primers were designed to bind exclusively to human sequences to demonstrate the presence of the human transdifferentiated cells in all transplanted mice at the end of the treatment (Fig. 4C). To verify the species-specificity of the qPCR assay, we set up reactions containing a known dilution ratio of both species, along with the qPCR primers that were chosen. Serial dilutions were used to explore the assay sensibility. Furthermore, human *PDX1* expression was detected in 1/3 (33%) of the mice that were analyzed with species-specific primer pairs by qPCR.

## Discussion

One of the most important findings in our work was that by manipulating culture conditions alone, fibroblasts from patients with T1D showed plasticity even to the point of crossing the boundaries of distinct developmental germ layers (mesoderm to endoderm). This fibroblast plasticity allowed the expression of pancreatic lineage transcription factors and genes, such as insulin, glucagon and somatostatin. These results suggest that the alteration of cell fate in diabetic patient skin fibroblasts were not only achieved by transgenesis [3] but also by manipulating culture conditions as well. The goal of altering the expression of genes using only chemical molecules has been well-documented in skin fibroblasts [22], foreskin fibroblasts [36] and pancreatic endocrine cells [37]. Additionally, skin fibroblasts were recently chemically reprogrammed toward a pluripotent state, which demonstrated extreme chemical capacities [23], [24]. In addition, the long-term stability and the lineage pancreatic-specific conversion in hESC or hiPSC depend exclusively on the introduction of chemical components in a specialized medium formulation [2]–[4], [38]. Together, these results indicate that chemical treatment alone can redirect the intrinsic plasticity of human skin fibroblasts into a pancreatic lineage. More recently, Pennarossa et al. [25] showed that it is also possible to convert adult non-diabetic fibroblasts into insulin secreting cells without the transgenesis step. However, this protocol includes a global demethylation step that uses the DNA methyltranferase inhibitor 5-azacytidine, which could encompasses several clinical risks, and does not use patient cells. Therefore, our results have shown that it is not necessary to use global demethylation agents to obtain a pancreatic phenotype, even in fibroblasts that are derived from diabetic patients, which allows the method to be much more straightforward for future clinical applications. This approach should provide a method to investigate T1D disease onset and will be helpful for testing therapeutic strategies.

However, although we observed reproducibility of the chemical transdifferentiation process in the fibroblasts from both diabetic patients (HF1 and HF2) and the healthy volunteer (HF0), there were differences in gene expression in the response of the protocol between replicates (Fig. 2A). This observation could be attributed to the random features of the reprogramming procedure, which has also been reported in other reprogramming protocols [6]. The activating mechanism of pancreatic gene lineage markers requires further investigation to turn this procedure into a non-stochastic process.

It has been suggested that transdifferentiation may require only a limited resetting in the DNA or chromatin, which is opposite to the extensive epigenetic resetting that is observed when reprogramming somatic cells into a pluripotent state [39]. The microarray analyses that were performed here demonstrated that our chemical transdifferentiation protocol created moderate changes on a transcriptome-wide scale. For this reason, we hypothesized that the transdifferentiation of fibroblasts to pancreatic-like cells require an amalgamate effect of upregulation in pancreatic-specific genes and of downregulation in fibroblast-specific genes but not an extensive change on a transcriptome-wide scale. Specifically, PDX1 is directly involved in the induction of pancreatic endoderm, whereas NGN3 induces the differentiation of pancreatic endocrine cells from epithelia progenitors during pancreatic differentiation. Recent studies support how the co-expression of these two factors leads to a greater increase in the *INS* gene than when only *PDX1* is upregulated [40]. Concurrently, the *INS+* chemically transdifferentiated in vitro fibroblasts showed *NGN3* and *PDX1* co-expression, which was observed by RT-PCR. *NGN3* was not detected in adult pancreas, in contrast with an evident detection of *PDX1*, which remains required for insulin production in β-cells. This result suggests that the transdifferentiated cells *NGN3*+ could be islet progenitors [41], [42]. Additionally, the expression of other pancreatic factors, such as ISL-1, which promotes the adult pancreatic islet cell proliferation, and PAX4, which is a marker of committed beta cell precursors [43], support the hypothesis that the chemical induction “switched” some transcription factors that are related to pancreatic and β-cell development in the reprogrammed insulin-expressing cells. Finally, many of the hormone-positive in vitro cells that were obtained here co-express glucagon, insulin and somatostatin, as shown previously [2], [35], which indicates a polyhormonal immature in vitro state.

Genomic DNA was isolated and bisulfate-treated, and the methylation percentage was analyzed by direct sequencing. Direct sequencing permitted the examination of the global DNA methylation profile in selected promoters and avoided the loss of existing epigenomes, which occurs when molecular cloning is used [44]. Based on the hypomethylation that was observed in the untreated fibroblasts, we hypothesized that epigenetic regulation, which is mediated by DNA methylation, is relaxed in fibroblasts compared with other specialized cells because changes in the epigenetic status in each cell linage could depend on its epigenetic background. Then, cell lineages under low epigenetic pressure during their phenotype acquisition, and hence poor commitment (as fibroblast lineage), can maintain a relaxed epigenetic profile. This is opposite to high-specialized cells that had strong epigenetic pressures to suppress or activate genes during their phenotype acquisition. The special hipomethylation status in fibroblasts, especially in *PDX1*, possibly contributes to the fibroblast pancreatic transdifferentiation capacity. Another committed type of cells that were used in this work did not display this relaxed methylation mosaic for the transcriptionally silenced genes. For example, complete methylation was observed for *OCT4* and frequently for *NANOG* in pancreatic human tissue (see Fig. 3B, C).

It is also well-known that epigenetic factors can regulate gene transcriptional events through methylation or acetylation over DNA and their associated histones inclusively in diabetes [45]–[48]. In the in vitro transdifferentiated *PDX1+* cells, the *PDX1* gene promoter was found demethylated, which is consistent with Kuroda et al. [49], who found significant differences in the methylation of the *INS* promoter gene during late pancreatic differentiation steps. This observation leads us to believe that chemical transdifferentiation is widely promising if the focus is on the discovery of a “specific” chemical inducer. For example, Ex4 ameliorates the symptoms of diabetes [50], [51] and has been reported as capable of causing pancreatic precursor cell differentiation into islet cells [52] and as activator of endogenous *PDX1* in rats [32]. Ex-4 prevents DNA methylation in the proximal promoter of *PDX1* by recruiting USF1 and PCAF transcription factors [32], which is consistent with the hypomethylation trend obtained from our transdifferentiated in vitro cells. This observation suggests that Ex-4 it is a key molecule for the activation of endogenous *PDX1*. The Ex-4 effects, as well as the epigenetic mosaic that was observed in the initial fibroblasts, possibly contribute to enhancing the access of activating elements to the chromatin. In addition, trough analyses of LINE 1 elements we observed that our chemical treatments did not affect the global methylation profiles, reinforcing that the identification of specific molecules can result in a non-global controlled epigenetic modifications. The elucidation of the precise mechanisms that are involved in our novel protocol is a major challenge for future studies.

To explore the scope of fibroblast plasticity, at the end of the treatment, a group of transdifferentiated in vitro cells were maintained for another 30 days in DMEM-Knockout with 10% SFB and antibiotics (without the pancreatic cocktail). Surprisingly, these cells returned to the fibroblast morphology and were negative for glucagon and somatostatin by RT-PCR (data not shown). These findings correspond with the study by Lysy et al. [22], in which these researchers demonstrated that chemically transdifferentiated fibroblasts are susceptible to losing the acquired phenotype after differentiation when replaced in a growth factor-free medium. In addition the absence of tumors obtained in the preliminary NUDE-mouse teratoma assays (data not shown) suggest that the nature of the remaining non-transdifferentiated and transdifferentiated cells, did not develop a pluripotent capacity after our chemical treatment.

To determine the in vitro functionality of this process, we measured C-peptide to demonstrate the de novo synthesis of insulin, avoiding any false-positive insulin from cells by uptake from the medium [2]. Although C-peptide was found in the *INS*-expressing transdifferentiated cells, along with glucagon, which was located by immunocytochemistry, the C-peptide detection was minimal by glucose induction, which resembled an immature pancreatic-like state in vitro. We can suggest that the transdifferentiated cells contained a certain quantity of C-peptide that was stored in vesicles, which did not receive the appropriate stimuli nor had specific membrane receptors to stimulate the vesicular trafficking to release C-peptide in the in vitro environment. As shown by D'Amour et al. [2] INS+ cells that were generated in vitro are devoid of mature β-cell characteristics, are not glucose responsive, and frequently coexpress other pancreatic hormones, such as pancreatic endocrine. The fully differentiated β-cells apparently require a sophisticated application scheme, which has not been reproduced thus far by any in vitro protocol. All these conclusions lead us to suggest that an in vivo transplant would be necessary to improve the islet survival and function of the transdifferentiated cells.

Pancreatic islets that are transplanted to the pancreas have a better revascularization than intra-hepatic islets and induce more gene expression changes [53], which explains why locating the transdifferentiated cells in their physiological environment could complement the differentiation process in vivo. Our data from in vivo assays support the observation that chemically transdifferentiated cells that were transplanted directly in the pancreas prevented the onset of extreme hyperglycemia (>600 mg/dl), which was reached by intrapancreatic transplanted sham and fibroblast controls. Additionally, human insulin serum was detected in nude mice with transdifferentiated transplanted cells, pre- and post-STZ induction. The multiple low-dose streptozotocin administration was intended to create a pancreas hypertrophy to condition the environment toward an islet regeneration and toward possible cell maturation, as well as to stimulate a selective survival phenotype in transplanted transdifferentiated cells [54]. Correct cell maturation is necessary for the correct expression of C-peptide and insulin [55]. The low insulin detection pre- and post-STZ indicated that some transplanted cells were nearly or in the maturation process to produce insulin and survived the cytotoxic treatment. Nevertheless, further studies must involve a correlation of insulin and C-peptide production with measurements of glycemia in different times during a prolonged experiment. However, the non-diabetic pancreas has approximately 1.0–1.7x10^6^ islets, which limit the β-cell mass to be equal to or above these parameters to positively correlate the insulin efficiency with the performance of a normal β-cell mass in the Langerhans region [56]. Kroon et al. [35], for instance, transplanted 1x10^7^ islet-like clusters to obtain a considerable amount of insulin in implanted diabetic mice serum. Therefore, considering the limitations that a transdifferentiated cell can go through in the in vivo model, such as lack of enough or a loss of transdifferentiated transplanted cells, we could determine that the human insulin quantity that was detected was significant within the 30-day treatment. Although it is true that glucose-stimulated Insulin-secreting (GSIS) test must be performed multiple times *in vivo*, our results are consistent with Sheng Ding and colleagues' research [57] who reported that small molecules improve the pancreatic induction in MEFs during different stages of the *in vitro* protocol and transplanted cells could ameliorate hyperglycemia in vivo. Finally, because none of the mice developed an acute pancreatitis or exacerbated inflammation, we considered this organ to be a good recipient for the reprogrammed cells. The pancreas appeared healthy to the naked eye, and the transdifferentiated cells did not form tumors in vivo or any related structure by one month post-transplantation in the diabetic mice, thus overcoming a critical obstacle to safe clinical translation.

Our findings represent a starting point for a long-term strategy that is intended to replace the damaged pancreas in insulin-dependent diabetes and that represents a novel strategy for encapsulating diabetes into a Petri dish for directly studying diabetes mechanisms and drug screening in T1D patient cells.

## Supporting Information

File S1 Table S1, S2 and S3; Figure S1.
(DOC)

File S2 Microarray supplementary information.
(XLS)
