# Supplementary material for: Skin Fibroblasts from Patients with Type 1 Diabetes (T1D) Can Be Chemically Transdifferentiated into Insulin-Expressing Clusters: A Transgene-Free Approach
Source: PLoS One. 2014 Jun 25;9(6):e100369. doi: 10.1371/journal.pone.0100369 (PMC4070975; doi:10.1371/journal.pone.0100369)
Supplement: File S1 — Table S1, S2 and S3; Figure S1. (DOC) [file pone.0100369.s001.doc]

**PLOS ONE**

**Electronic Supplementary Material 1**

**Skin Fibroblasts from Patients with Type 1 Diabetes (T1D) can be Chemically Transdifferentiated into Insulin-Expressing Clusters: a Transgene-Free Approach**

F. Pereyra-Bonnet et al.


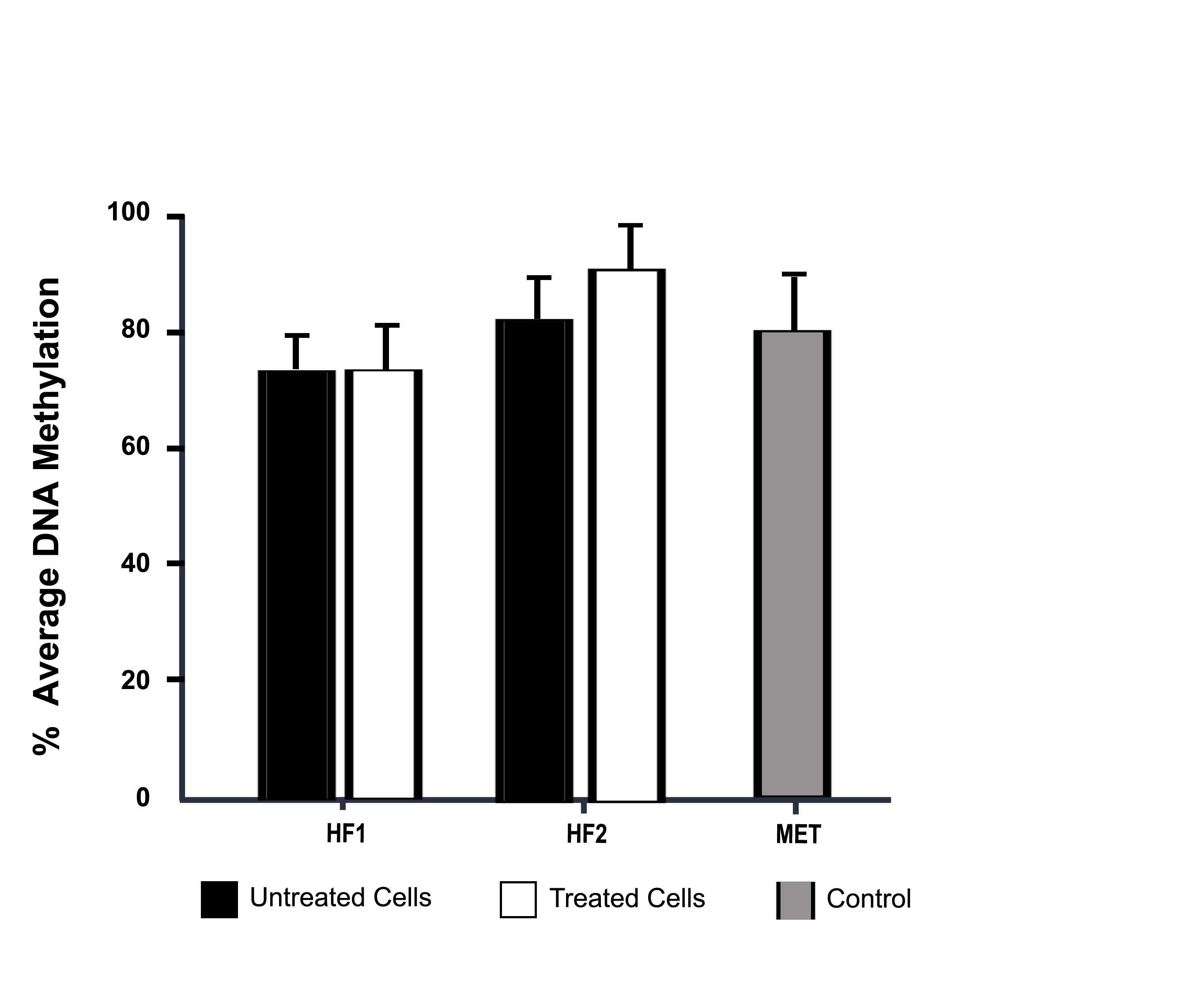


Figure S1. Methylation levels using LINE 1 as a global methylation marker in human fibroblast before and after chemical treatment. A fully methylated human DNA is included as a control (MET). Data are presented as the mean percentage of methylation in the LINE 1 promoter of various replicates (n=3-13). CpG included in measurement were number 257, 271, 286 and 295 using as reference LINE 1 promoter sequence (X58075). No changes on methylation levels were found (*P*>0.05). Error bars represent standard deviations.

Table S1. Primers used for RT-PCR, quantitative PCR (qPCR) and bisulphyte (PCRbis)

|  | Forward primer (5’ to 3’) | Reverse primer (5’ to 3’) |
| --- | --- | --- |
| *RT-PCR* |  |  |
| *INS* | GCAGCCTTTGTGAACCAACACC | TGTTCCACAATGCCACGCTTC |
| *GCG* | ACTTTGTGGCTGGATTATTTGT | GTTTGGCAATGTTATTCCTGTT |
| *STT* | TTTAGGAGCGAGGTTCGG | TCAGGTTCCAGGGCATCA |
| *NGN3* | TCAACTCGGCACTGGACG | AGGGAGAAGCAGAAGGAACAA |
| *PDX1* | CCCATGGATGAAGTCTACC | GTCCTCCTCCTTTTTCCAC |
| *qPCR* |  |  |
| *INS* | GCAGCCTTTGTGAACCAACA | CGGGTCTTGGGTGTGTAGAAGAAG |
| *GCG* | ACTTTGTGGCTGGATTATTTGT | GTTTGGCAATGTTATTCCTGTT |
| *ASPN* | CCACCAACTTTATTGGAGCTTCAC | CGTGGTATGTTAGCAAGACTCCC |
| *MEOX2* | TCTCACCAGACTGAGGCGATAC | TCCACTTCATCCGCCTGTTTTGG |
| *SRY* | ACAGGGATGACTGTACGAA | CTTTGTCCAGTGGCTGTAG |
| *PDX1* | GTCCTGGAGGAGCCCAAC | TACTTGTTGAATAGGAACT |
| *Β-Actin* | CCCTTGCCATCCTAAAAGCC | TGCTATCACCTCCCCTGTGT |
| *B-Actin mouse* | GCCCAGAGCAAGAGATGTA | AGAAGGTGTGGTGCCAGAT |
| *PCR bis* |  |  |
| *PDX1* | AATTTAGTTGAGAGAGAAAATTGGA | CCATATACAAACACRCAAAAA |
| *OCT4* | GTTAGAGGTTAAGGTTAGTGGGTG | AAACCTTAAAAACTTAACCAAATCCA |
| *NANOG* | TGGTTAGGTTGGTTTTAAATTTTTG | AACCCACCCTTATAAATTCTCAATTA |
| *LINE1* | TTTTGAGTTAGGTGTGGGATATA | AAAATCAAAAAATTCCCTTTC |
| *LINE1sec* | AGTTAGGTGTGGGATATAGT |  |

Table S2. DNA fingerprint of parental fibroblasts and treated cells

|  | HF1 | | HF2 | |
| --- | --- | --- | --- | --- |
|  | parental | transdifferentiated | Parental | transdifferentiated |
| HUMCSF1PO | 11/12 | 11/12 | 12/13 | 12/13 |
| HUMTPOX | 8/10 | 8/10 | 8/9 | 8/9 |
| HUMTH01 | 6/9.3 | 6/9.3 | 8/9.3 | 8/9.3 |
| HUMF13A01 | 6/7 | 6/7 | 7/7 | 7/7 |
| HUMESFPS | 10/11 | 10/11 | 10/11 | 10/11 |
| HUMVWFA31 | 17/18 | 17/18 | 15/17 | 15/17 |
| D16S539 | 10/13 | 10/13 | 10/12 | 10/12 |
| D7S820 | 8/11 | 8/11 | 8/10 | 8/10 |
| D13S317 | 11/11 | 11/11 | 10/11 | 10/11 |

Representative results for 9 polymorphic short tandem repeat DNA loci are show here

Table S3. Set of key genes up or dow-regulated after chemical treatment

|  | **Signal** | | | | | |
| --- | --- | --- | --- | --- | --- | --- |
|  | **HF1** | | | **HF2** | | |
| ***GENES*** | **Untreated** | **Treated** | **Fold change T/C** | **Untreated** | **Treated** | **Fold change**  **T/C** |
| ***Development*** |  |  |  |  |  |  |
| BMP4 | 1735.066 | 927.0196 | **-1.78** | 1364.255 | 492.6393 | **-2.60** |
| ACVR2A | 2847.094 | 1116.782 | **-2.42** | 4966.925 | 1210.584 | **-3.90** |
| OSR1 | 7897.578 | 15667.03 | **2.27** | 3735.061 | 14697.32 | **4.07** |
| TBX3 | 814.182 | 1372.188 | **1.19** | 432.0154 | 1610.413 | **1.16** |
| ***Endocrine*** |  |  |  |  |  |  |
| MAFB | 852.8494 | 2304.097 | **2.84** | 2722.784 | 5421.531 | **2.07** |
| KLF10 | 257.6819 | 1100.459 | **4.54** | 448.6744 | 1126.161 | **2.65** |
| NES | 227.1197 | 454.3278 | **2.00** | 264.2184 | 511.1486 | **1.93** |
| ***Bone development*** |  |  |  |  |  |  |
| BMP2 | 354.5111 | 2810.964 | **8.29** | 776.9 | 1075.601 | **1.46** |
| BMP4 | 1735.066 | 927.0196 | **-1.78** | 1364.255 | 492.6393 | **-2.60** |
| BMP6 | 386.3282 | 204.1304 | **-1.8** | 1112.103 | 322.5734 | **-3.26** |
| THRA | 8834.126 | 2630.428 | **-3.0** | 12418.72 | 2364.833 | **-5.11** |
| SPARC | 28900 | 27671.26 | **1.14** | 36788.02 | 14401 | **-2.54** |
| ***Neuron development*** |  |  |  |  |  |  |
| SEMA5A | 3130.661 | 1352.692 | **-2.03** | 6958.275 | 2352.236 | **-3.38** |
| SEMA3B | 652.5526 | 532.7869 | **-1.16** | 480.6773 | 216.8522 | **-2.10** |
| SEMA3A | 1324.137 | 1467.901 | **1.17** | 843.0541 | 1706.984 | **2.13** |
| ROBO3 | 882.6411 | 1004.479 | **1.20** | 608.2202 | 1196.633 | **2.08** |
| NGF | 2027.638 | 471.6562 | **-4.08** | 3221.198 | 1327.203 | **-2.30** |
| ***Muscle cell development and muscle cell markers*** |  |  |  |  |  |  |
| ACTG1 | 4537.823 | 11570.56 | **2.85** | 6506.5 | 12038.03 | **1.96** |
| ACTC1 | 169.1638 | 319.4217 | **2.04** | 402.5369 | 224.333 | **-1.70** |
| TPM1 | 4850.129 | 4527.546 | **-1.12** | 6926.705 | 1590.627 | **-4.52** |
| CDH15 | 151.22 | 132.3149 | **0.87** | 119.5324 | 149.2521 | **1.25** |
| CD34 | 614.7759 | 247.7223 | **-2.36** | 1741.683 | 231.1774 | **-7.16** |
| ***Skin development*** |  |  |  |  |  |  |
| COL3A1 | 32437.5 | 13626.68 | **-1.99** | 45592.39 | 32437.5 | **-2.90** |
| COL1A2 | 62232.02 | 42069.26 | **-1.27** | 61975.4 | 23551.47 | **-1.70** |
| COL1A1 | 39191.43 | 17326.63 | **-1.91** | 51123.93 | 12787.67 | **-3.97** |
| COL5A2 | 10972.07 | 5991.112 | **-1.59** | 25406.67 | 10751.92 | **-2.28** |
| ADAMTS2 | 1284.892 | 541.7427 | **-2.25** | 1080.623 | 508.2522 | **-2.00** |
| ***Extracellular matrix*** |  |  |  |  |  |  |
| COL8A1 | 2480.151 | 299.0494 | **-7.9** | 16653.33 | 541.0804 | **-29.38** |
| FMOD | 759.7853 | 321.8079 | **-2.24** | 5329.169 | 1223.9 | **-4.12** |
| ELN | 884.2727 | 542.041 | **-1.54** | 1655.42 | 347.0803 | **-4.51** |
| TNC | 7137.219 | 4117.901 | **-1.47** | 9910.19 | 27624.29 | **3.43** |
| LAMB2 | 8506.021 | 5421.308 | **-1.39** | 9031.922 | 4111.726 | **-2.14** |
| LAMB3 | 260.1513 | 341.7419 | **1.39** | 232.3912 | 569.2547 | **2.51** |
| ***Cell adhesion*** |  |  |  |  |  |  |
| CDH6 | 821.5555 | 243.5551 | **-3.20** | 415.6467 | 179.8999 | **-2.20** |
| CD9 | 893.2396 | 3807.646 | **4.54** | 136.4263 | 737.9891 | **5.40** |
| FBLN5 | 10794.25 | 5508.819 | **-1.70** | 12691.65 | 3206.777 | **-3.84** |
| ***Cell cycle*** |  |  |  |  |  |  |
| BCAT1 | 571.6075 | 168.8199 | **-3.23** | 912.2287 | 298.8497 | **-2.90** |
| C13ORF15 | 3561.411 | 9460.042 | **2.90** | 11888.59 | 16476.26 | **1.40** |
| MYC | 3232.452 | 6026.554 | **2.04** | 4038.953 | 11621.57 | **3.00** |
| WEE1 | 1986.476 | 783.2482 | **-2.41** | 3235.484 | 1460.578 | **-2.10** |
| ***Chromatin modification and organization*** |  |  |  |  |  |  |
| CHD1 | 969.2736 | 1327.384 | **1.43** | 955.8207 | 2213.501 | **2.43** |
| RRS1 | 674.7177 | 1573.29 | **2.45** | 851.6713 | 1953.557 | **2.41** |
| CPA4 | 955.7348 | 311.3683 | **-2.94** | 479.8844 | 188.1608 | **-2.42** |
| HMGA1 | 5405.944 | 5069.03 | **1.04** | 2094.859 | 9734.585 | **4.88** |
| ASF1B | 429.4221 | 178.6204 | **-2.23** | 310.7863 | 210.0507 | **-1.40** |
